# SARS-CoV-2 Is Restricted by Zinc Finger Antiviral Protein despite Preadaptation to the Low-CpG Environment in Humans

**DOI:** 10.1128/mBio.01930-20

**Published:** 2020-10-16

**Authors:** Rayhane Nchioua, Dorota Kmiec, Janis A. Müller, Carina Conzelmann, Rüdiger Groß, Chad M. Swanson, Stuart J. D. Neil, Steffen Stenger, Daniel Sauter, Jan Münch, Konstantin M. J. Sparrer, Frank Kirchhoff

**Affiliations:** a Institute of Molecular Virology, Ulm University Medical Center, Ulm, Germany; b Department of Infectious Diseases, School of Immunology and Microbial Sciences, King's College London, London, United Kingdom; c Institute of Medical Microbiology and Hygiene, Ulm University Medical Center, Ulm, Germany; University of Massachusetts Medical School; Columbia University/HHMI

**Keywords:** COVID-19, CpG suppression, SARS-CoV-2, ZAP, evolution, interferon

## Abstract

Recent evidence shows that severe acute respiratory syndrome coronavirus 2 (SARS-CoV-2) is sensitive to interferons (IFNs). However, the most effective types of IFNs and the underlying antiviral effectors remain to be defined. Here, we show that zinc finger antiviral protein (ZAP), which preferentially targets CpG dinucleotides in viral RNA sequences, restricts SARS-CoV-2. We further demonstrate that ZAP and its cofactors KHNYN and TRIM25 are expressed in human lung cells. Type I, II, and III IFNs all strongly inhibited SARS-CoV-2 and further induced ZAP expression. Comprehensive sequence analyses revealed that SARS-CoV-2 and its closest relatives from horseshoe bats showed the strongest CpG suppression among all known human and bat coronaviruses, respectively. Nevertheless, endogenous ZAP expression restricted SARS-CoV-2 replication in human lung cells, particularly upon treatment with IFN-α or IFN-γ. Both the long and the short isoforms of human ZAP reduced SARS-CoV-2 RNA expression levels, but the former did so with greater efficiency. Finally, we show that the ability to restrict SARS-CoV-2 is conserved in ZAP orthologues of the reservoir bat and potential intermediate pangolin hosts of human coronaviruses. Altogether, our results show that ZAP is an important effector of the innate response against SARS-CoV-2, although this pandemic pathogen emerged from zoonosis of a coronavirus that was preadapted to the low-CpG environment in humans.

## Abstract (precis)

Although interferons inhibit SARS-CoV-2 and have been evaluated for treatment of coronavirus disease 2019 (COVID-19), the most effective types and antiviral effectors remain to be defined. Here, we show that IFN-γ is particularly potent in restricting SARS-CoV-2 and in inducing expression of the antiviral factor ZAP in human lung cells. Knockdown experiments revealed that endogenous ZAP significantly restricts SARS-CoV-2. We further show that CpG dinucleotides which are specifically targeted by ZAP are strongly suppressed in the SARS-CoV-2 genome and that the two closest horseshoe bat relatives of SARS-CoV-2 show the lowest genomic CpG content of all coronavirus sequences available from this reservoir host. Nonetheless, both the short and long isoforms of human ZAP reduced SARS-CoV-2 RNA levels, and this activity was conserved in horseshoe bat and pangolin ZAP orthologues. Our findings indicating that type II interferon is particularly efficient against SARS-CoV-2 and that ZAP restricts this pandemic viral pathogen might promote the development of effective immune therapies against COVID-19.

## INTRODUCTION

Severe acute respiratory syndrome coronavirus 2 (SARS-CoV-2), the causative agent of coronavirus disease 2019 (COVID-19), was first detected in humans in Wuhan, China, at the end of 2019 and rapidly spread in the human population, causing a devastating pandemic (1). As of 16 September 2020, almost 30 million infections with SARS-CoV-2 around the globe have been confirmed, and the virus had caused almost 1 million deaths (https://coronavirus.jhu.edu/map.html). While SARS-CoV-2 usually causes no or relatively mild respiratory infections in younger individuals, it regularly results in severe respiratory disease and death in the elderly and in people with specific medical conditions, such as asthma, heart diseases, diabetes, or severe obesity (2). SARS-CoV-2 is spreading substantially more efficiently than the first zoonotic highly pathogenic coronavirus (SARS-CoV), which emerged in 2002 and infected about 8,000 individuals (3, 4). Despite its rapid global spread, SARS-CoV-2 seems to be more susceptible to inhibition by type I interferons (IFNs) than SARS-CoV (5). Consequently, type I IFNs are currently being considered for treatment of COVID-19 (6).

Treatment with IFNs induces the expression of hundreds of cellular IFN-stimulated genes (ISGs), and it is currently unknown which of these genes contribute to IFN-inducible restriction of SARS-CoV-2 replication. However, antiviral factors may exert strong selection pressure that results in specific viral properties that provide hints with respect to efficient IFN-mediated immune responses. For example, it has long been known that coronaviruses display marked suppression of CpG dinucleotides (7) and recent evidence shows that this is also the case for SARS-CoV-2 (8). At least in part, this CpG suppression may be driven by the zinc finger antiviral protein (ZAP) that restricts numerous viral pathogens (9) and specifically targets CpG-rich RNA sequences that are underrepresented in the human transcriptome (10).

Coronaviruses (CoVs) are found in numerous animal species, such as bats, swine, cattle, horses, camels, cats, dogs, rodents, rabbits, ferrets, civets, pangolins, birds, and snakes (11, 12). They have successfully crossed the species barrier to humans at least seven times, and it is thought that all human CoVs (hCoVs) originate from ancestral bat viruses, although intermediate hosts frequently facilitated viral zoonoses (13). Four human coronaviruses are associated with seasonal common colds. Two of these (CoV-229E and CoV-OC43) were identified more than 60 years ago and are relatively well adapted to humans. Two other coronaviruses associated with a range of respiratory symptoms were identified in 2004 (CoV-NL63) and 2005 (CoV-HKU1) (14, 15). While these strains usually cause mild respiratory diseases, three additional coronaviruses responsible for severe lung disease emerged from viral zoonoses in the last 20 years. In 2003, SARS-CoV was identified as the causative agent of severe acute respiratory syndromes (SARS) with ∼10% mortality (16). The highly lethal pathogen Middle East respiratory syndrome-CoV (MERS-CoV) appeared in 2012 and is associated with case fatality rates of almost 40% (17). The current SARS-CoV-2 shows a lower infection fatality rate (<1%) but is spreading at enormous speed. While the direct animal precursor remains to be identified, close relatives of SARS-CoV-2 have been detected in horseshoe bats (1, 18) and pangolins (19, 20).

To define selection pressures on SARS-CoV-2 and other coronaviruses, we examined CpG frequencies and distributions in all seven human viruses and their closest animal counterparts. We found that CpG dinucleotides are generally suppressed and observed a trend toward lower CpG frequencies in hCoVs compared to their nonhuman relatives. In agreement with recent *in silico* studies (8, 21), SARS-CoV-2 showed stronger CpG suppression than SARS-CoV and MERS-CoV, albeit with substantial variation across its genome (22). Remarkably, the closest bat relatives of SARS-CoV-2 display the strongest CpG suppression of all coronaviruses available from this reservoir host. We found that endogenous ZAP expression nevertheless significantly contributes to inhibition of SARS-CoV-2 by interferons. Although a relatively low frequency of CpG dinucleotides may have facilitated the spread of SARS-CoV-2, it clearly does not render this pandemic pathogen fully resistant to ZAP-mediated restriction.

## RESULTS

### SARS-CoV-2 and its closest bat relatives show strong CpG suppression.

**FIG 1:**
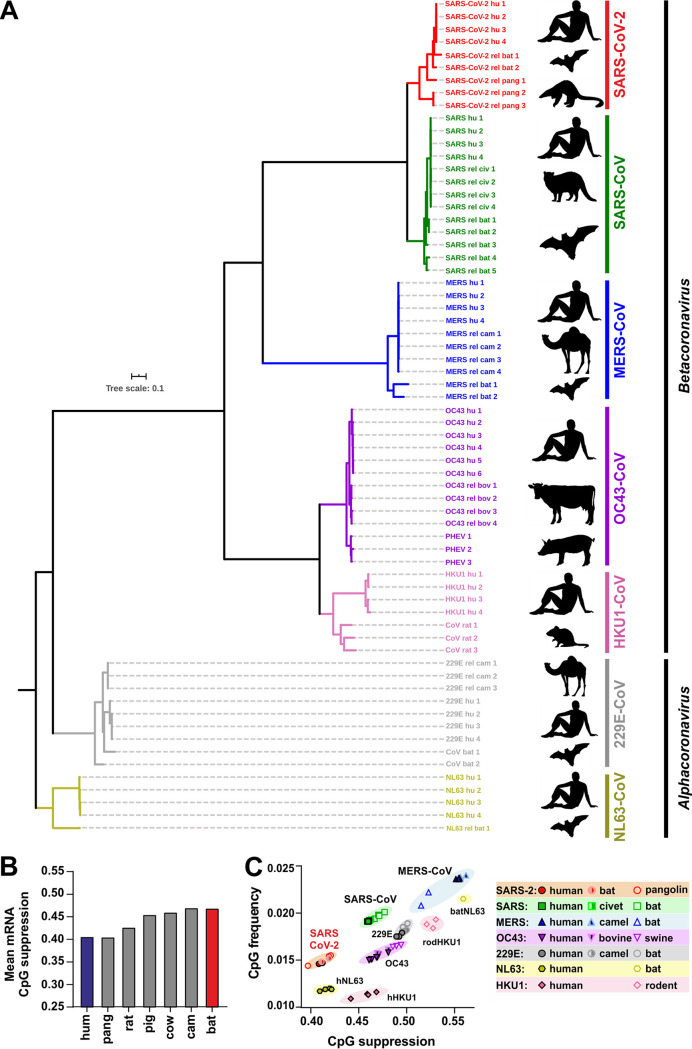
Phylogenetic relationship between human coronaviruses and their animal relatives and CpG suppression in pathogens and their hosts. (A) Distance-based relationship inference based on representative full-genome nucleotide sequences of human-infecting SARS-CoV-2, SARS-CoV, MERS-CoV, HKU1-CoV, OC43-CoV, NL63-CoV, and 229E-CoV strains and their closest known animal relatives. Black symbols to the right indicate the viral host (human, horseshoe bat, pangolin, civet, camel, rat, pig, or cattle). (B) Mean CpG suppression (i.e., number of observed CpGs normalized to expected CpGs based on sequence length and GC content) in mRNAs of the indicated host species (human [hum], pangolin [pang], rat, pig, cow, camel [cam], or horseshoe bat [bat]). (C) CpG frequency (number of CpGs normalized to sequence nucleotide length) and suppression (number of observed CpGs normalized to expected CpGs based on sequence length and GC content) in human coronavirus genomes and their closest animal-infecting relatives.

To determine the frequency and distribution of CpG dinucleotides and to identify possible differences in the levels of suppression, we analyzed 67 genomes representing the seven human coronaviruses (hCoVs) and their closest animal relatives (Fig. 1A; see also Table S1 in the supplemental material). Direct animal precursors or close relatives of emerging human SARS-CoV-2, MERS-CoV-2, and SARS-CoV-2, as well as seasonal hCoV-229E and hCoV-OC43, have been previously identified (Fig. 1A). In contrast, the closest known animal relatives of hCoV-HKU1 and hCoV-NL63 found in rats and bats show only ∼75% sequence identity to the corresponding human coronaviruses (Table S1), indicating a long history of evolutionary divergence (23). Even though the immediate animal precursors are not always known, it is assumed that all seven hCoVs originated from bats, mice, or domestic animals, where bats, which harbor an enormous diversity of CoVs, represent the reservoir host (11, 24).

10.1128/mBio.01930-20.5 TABLE S1 Features of coronavirus sequences analyzed. Download Table S1, DOCX file, 0.03 MB. Copyright © 2020 Nchioua et al. 2020 Nchioua et al. This content is distributed under the terms of the Creative Commons Attribution 4.0 International license.

RNA viruses infecting vertebrates are known to mimic the CpG suppression of their hosts, and increased viral CpG suppression following zoonotic transmission has been proposed to represent an important human-specific adaptation (25). To assess whether zoonotic transmission of CoVs to humans might increase the selection pressure against CpGs, we first analyzed the levels of CpG suppression in the reservoir bat and in intermediate and human hosts. While the human genome and transcriptome have been extensively studied and the data have undergone multiple quality checks, the transcript data sets of other species mostly contain predicted mRNA sequences and could be biased by the presence of poor-quality transcripts or modeling errors. We therefore removed mRNA transcripts containing stretches of non-ATCG bases from the analysis and also quantified length-dependent CpG suppression to determine if the differences in the average suppression levels were consistent across data sets that included at least 40,000 transcripts of each species. Overall, the levels of CpG suppression vary and inversely correlate with the length of the cellular RNAs (see Fig. S1A in the supplemental material), most likely due to the presence of regulatory elements in the 5′ untranslated region (UTR) (26, 27). On average, however, CpG suppression was found to be more pronounced in humans than in the horseshoe bat, while the rat, pig, and cow hosts showed an intermediate phenotype, with the levels seen with camels being similar to those seen with horseshoe bats and the levels seen with pangolins being similar to those seen with humans (Fig. 1B). Analysis of the genomes of human coronaviruses and their animal relatives revealed that all of them show significant CpG suppression, although to differing extents (CpG frequency, 0.39 to 0.67; Fig. 1C). MERS-CoV, associated with the highest host mortality but also with the most limited spread, was the least-CpG-suppressed human coronavirus. In contrast, SARS-CoV-2 showed the strongest CpG suppression, approximating the levels of suppression existing in its human host (Fig. 1C; see also Table S1). We also examined the frequencies of UpAs since some RNA viruses show biased frequencies in this dinucleotide (28), and it was previously reported that ZAP may also target UpA dinucleotides (29). We found that coronaviruses show UpA suppression, although to only a small extent (0.71 to 0.97; Fig. S1B).

10.1128/mBio.01930-20.1 FIG S1 Length-dependent CpG suppression in host mRNAs and UpA content of human and animal coronaviruses. (A) CpG suppression in host mRNA transcripts grouped by average length as indicated. (B) UpA frequency (number of UpAs normalized to sequence nucleotide length) and suppression (number of observed UpAs normalized to expected number of UpAs based on sequence length and UpA content) in human-infecting coronavirus genomes and their closest animal-infecting relatives. (C) UpA frequency and suppression of sequenced bat coronaviruses available in NCBI database (*n* = 182). Close relatives of SARS-CoV-2 are shown in bright (RaTG13) and dark (RmYN02) red. Each point represents one viral strain. Download FIG S1, TIF file, 0.4 MB. Copyright © 2020 Nchioua et al. 2020 Nchioua et al. This content is distributed under the terms of the Creative Commons Attribution 4.0 International license.

**FIG 2:**
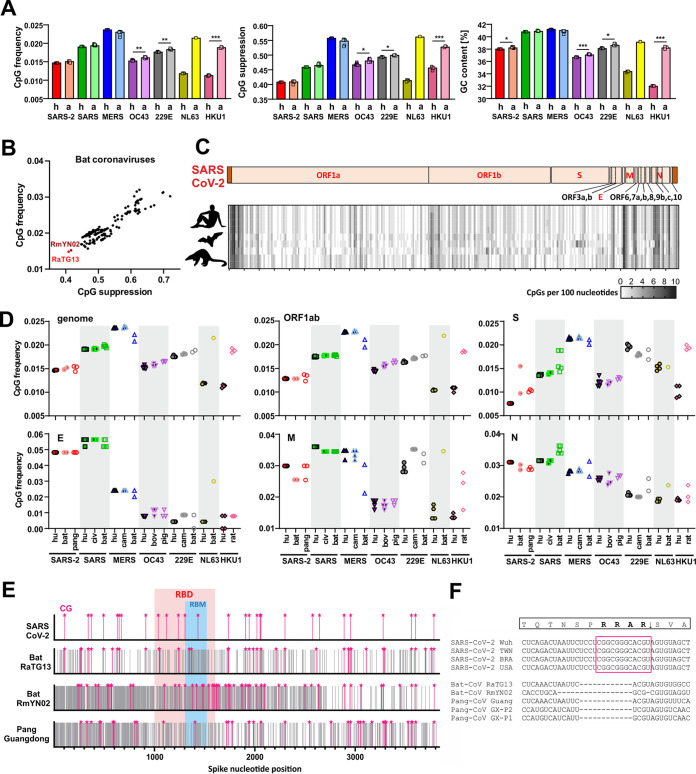
CpG content and distribution in human coronaviruses and their animal relatives. (A) CpG frequency, suppression, and GC content in the genomes of human (h)-infecting and animal (a)-infecting coronaviruses. (B) CpG frequency and suppression of sequenced bat coronaviruses available in NCBI database (*n* = 182); close relatives of SARS-CoV-2 are shown in bright (RaTG13) and dark (RmYN02) red. Each point represents one viral strain. (C) Heat map showing the number of CpG dinucleotides, ranging from 0 (white) to 10 (black), within 100-bp sliding windows of aligned genomic sequences of SARS-CoV-2 and its closest relatives infecting horseshoe bats and pangolins. The genome organization diagram at the top represents that of the human virus. (D) CpG frequency in major genes of human and related animal coronaviruses. These genes encode viral polyproteins (ORF1ab), envelope (E), spike (S), nucleocapsid (N), and matrix (M) proteins. bov, bovine; civ, civet. (E) Schematic representation of spike nucleotide sequences of SARS-CoV-2 and its closest relatives showing the relative positions of CpGs (pink stars), receptor binding domains (RBD), and receptor binding motifs (RBM). Gray lines indicate nucleotide mismatches compared to aligned SARS-CoV-2 spike. (F) Insertion in spike of SARS-CoV-2 introducing a novel furin-cleavage site after an RRAR motif.

To assess whether the selection pressure against viral CpGs increases after zoonotic transmission, we compared CpG suppression and frequency data, as well as the levels of GC content of hCoVs and their closest known animal relatives. Circulating hCoVs associated with seasonal colds showed significantly lower CpG frequencies and stronger suppression than their closest animal relatives, while this was not the case for the highly pathogenic SARS-CoVs and MERS-CoVs (Fig. 1C; see also Fig. 2A). SARS-CoV and MERS-CoV showed higher genomic GC content than the remaining CoVs, which explains why they displayed higher CpG frequencies at the same level of CpG suppression (Fig. 2A). SARS-CoV-2 and its closest relatives from horseshoe bats and pangolins showed stronger CpG suppression than most other CoVs. Consequently, their CpG frequencies are similar to those found in minimally pathogenic CoVs and lower than those found in SARS-CoV and MERS-CoV as well as in their relatives from bats, camels, and civet cats (Fig. 1C; see also Fig. 2A). These results raised the possibility that SARS-CoV-2 originated from a zoonotic virus showing a particularly low frequency of CpG dinucleotides. Indeed, the two closest animal relatives of SARS-CoV-2 (RaTG13 and RmYN02) showed markedly lower CpG frequencies than all 180 of the remaining bat viruses available for analysis (Fig. 2B). In contrast, their levels of UpA suppression were modest and close to the average observed for bat coronaviruses (Fig. S1C).

Coronavirus genomes differ in length (Table S1) and in the presence of specific accessory genes (Fig. S2). Thus, we generated individual CpG distribution heat maps for each group of hCoVs and their animal counterparts (Fig. 2C; see also Fig. S2) and compared CpG frequencies in the major viral genes (Fig. 2D). On average, SARS-CoV-2 showed substantially lower CpG frequencies (0.014) than SARS-CoV (0.019) and MERS-CoV (0.024) (Fig. 2A). However, we observed fluctuation between individual genes. While CpGs were strongly suppressed in the large ORF1a/b and Spike (S) open reading frames (ORFs), both SARS-CoV and SARS-CoV-2 showed high numbers of CpGs in the 3′ regions of their genomes and the 5′ UTR (Fig. 2C; see also Fig. S2). Consequently, they displayed higher CpG frequencies than other CoVs in the E (envelope) and (to a lesser extent) N (nucleocapsid) coding regions (Fig. 2D). However, the E gene encompasses just 228 to 267 bp. Thus, small changes in CpG numbers have a large impact on their frequency.

10.1128/mBio.01930-20.2 FIG S2 CpG distribution in the genomes of human and animal coronaviruses. Data represent CpG frequency in human and animal coronavirus genomes. The color shade indicates the number of CpGs per 100 nucleotides (0 [white] to 10 [black]). Genome organization diagrams at the top represent the reference human virus as indicated. Download FIG S2, TIF file, 2.8 MB. Copyright © 2020 Nchioua et al. 2020 Nchioua et al. This content is distributed under the terms of the Creative Commons Attribution 4.0 International license.

Notably, a region in the Spike gene of the bat CoV-RmYN02 strain that is otherwise closely related to SARS-CoV-2 (18) encoding amino acid residues involved in interaction with the viral ACE2 receptor showed low nucleotide identity and a much higher frequency of CpGs than SARS-CoV-2 (Fig. 2E). In addition, a small insertion that is present in SARS-CoV-2 Spike but not in its relatives from horseshoe bats and pangolins introduced not only a potential furin cleavage site but also an additional clustered CpG motif that may be targeted by ZAP (Fig. 2F). Altogether, our results support the idea that the selective pressure against CpGs is increased upon zoonotic transmission from bats and most intermediate hosts to humans. This indicates that the differences between hCoVs and their animal relatives may reflect different degrees of adaptation. At least in part, however, SARS-CoV-2 may have been preadapted to the low-CpG environment in humans because its closest known counterparts from bats contain an unusually low frequency of CpG dinucleotides.

### All three types of IFN inhibit SARS-CoV-2 and induce ZAP expression.

Our sequence analyses indicated that successful zoonotic transmission of CoVs to humans is associated with increased selection pressure for CpG suppression. To assess whether the antiviral factor ZAP might be the driving force behind this, we first examined whether ZAP is expressed in viral target cells. Western blot analyses of the human epithelial lung cancer cell lines Calu-3 and A549 that are commonly used in SARS-CoV-2 research (30, 31), as well as of primary human lung fibroblasts, showed that all of these constitutively express the short and long isoforms of ZAP (Fig. S3A to C). Treatment with TNF-α as well as IFN-α, IFN-β, and IFN-γ had modest effects on expression of the long isoform of ZAP but usually enhanced expression of the short isoform. IFN-γ exhibited the most striking effects and increased expression of the short isoform of ZAP [ZAP(S)] up to 8-fold (Fig. S3, right panels). We also examined expression of TRIM25 and KHNYN because ZAP itself does not possess RNase activity and because it has been reported that these cofactors are critical for effective viral restriction (32–34). TRIM25 and KHNYN were constitutively expressed in Calu-3 and A549 cells, and the former was further induced by IFNs. KHNYN expression was not detectable in primary lung fibroblasts (Fig. S3C).

10.1128/mBio.01930-20.3 FIG S3 Expression and IFN induction of ZAP and its cofactors KHNYN and TRIM25. (A to C) Expression of endogenous ZAP, KHNYN, and TRIM25 in (A) Calu-3, (B) A549, and (C) primary human lung fibroblasts (NHLF) after stimulation with the indicated cytokines. An immunoblot of whole-cell lysates stained with anti-ZAP, anti-KHNYN, or anti-TRIM25 is shown. GAPDH served as a protein loading control. Relative levels of expression of the long (L) isoforms or short (S) isoforms of ZAP, KHNYN, and TRIM25, normalized to unstimulated cells set as 100%, are indicated in the right panels. KHNYN was not quantified for NHLF because the expression levels were too low. Download FIG S3, TIF file, 1.5 MB. Copyright © 2020 Nchioua et al. 2020 Nchioua et al. This content is distributed under the terms of the Creative Commons Attribution 4.0 International license.

**FIG 3:**
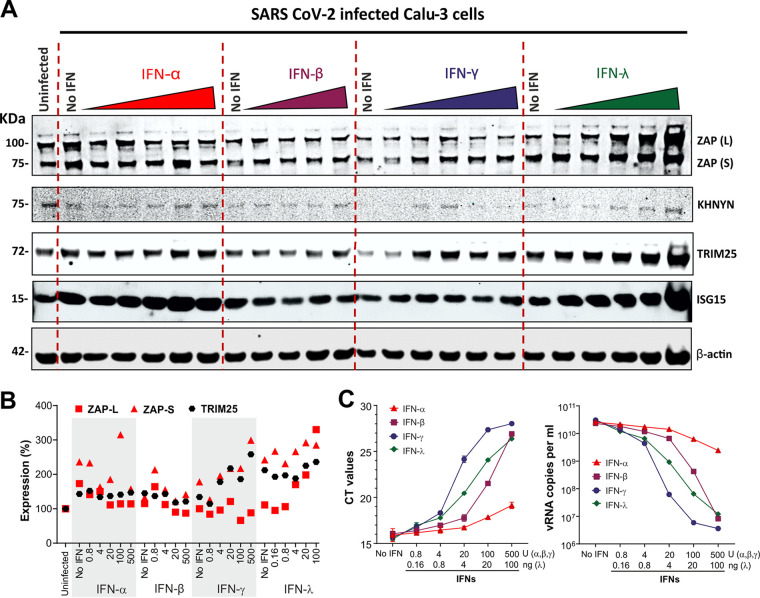
Inhibition of SARS-CoV-2 by different types of IFN. (A) Endogenous expression of ZAP and its cofactors KHNYN and TRIM25 in Calu-3 cells that were left untreated and/or uninfected or were treated with the amounts of IFNs indicated in panel B and infected with SARS-CoV-2. Whole-cell lysates were immunoblotted and stained with anti-ZAP, anti-KHNYN, anti-TRIM25, anti-ISG15 and anti-β-actin. (B) Relative expression levels of the long (L) or short (S) isoforms of ZAP and TRIM25 normalized to unstimulated cells set as 100%. The data were derived from the Western blots shown in panel A. (C) Raw RT-qPCR threshold cycle (*C_T_*) values (left) and corresponding SARS-CoV-2 RNA copy numbers per ml (right) in the supernatants of Calu-3 cells were determined as described for panel A. *n* = 1 in technical duplicate. Shown are mean values ±SD.

IFNs are currently being evaluated for the treatment of COVID-19 (6). However, it is under debate which type of IFN is most effective against SARS-CoV-2 (35). To determine which IFNs are most potent in inhibiting SARS-CoV-2 and in inducing ZAP, we performed titration experiments using type I (α/β), II (γ), and III (λ) IFNs. We selected Calu-3 cells for these experiments because they are highly susceptible to SARS-CoV-2 infection (30, 36), express ZAP and its cofactors (Fig. S3A), and seemed most suitable for subsequent small interfering RNA (siRNA) knockdown (KD) studies. Treatment with the different types of IFNs was associated with modest to marked increases in ZAP expression, and IFN-α and IFN-λ strongly induced ISG15 used as control of ISG stimulation (Fig. 3A and B). Determination of virus yields by real-time quantitative PCR (RT-qPCR) showed that IFN-γ reduced virus production by almost 4 orders of magnitude at 100 U/ml (Fig. 3C). IFN-β and IFN-λ were also highly potent against SARS-CoV-2, whereas IFN-α showed only modest inhibitory activity. Altogether, our data add to the recently reported evidence (5, 37) indicating that IFNs are highly effective against SARS-CoV-2. However, they also revealed that at least in Calu-3 cells, IFN-γ is particularly effective and IFN-α only weakly active against SARS-CoV-2. In addition, our results show that ZAP and its cofactors are expressed in SARS-CoV-2 target cells and agree with a potential role of ZAP in the antiviral effect of the various IFNs.

### Endogenous ZAP expression restricts SARS-CoV-2.

**FIG 4:**
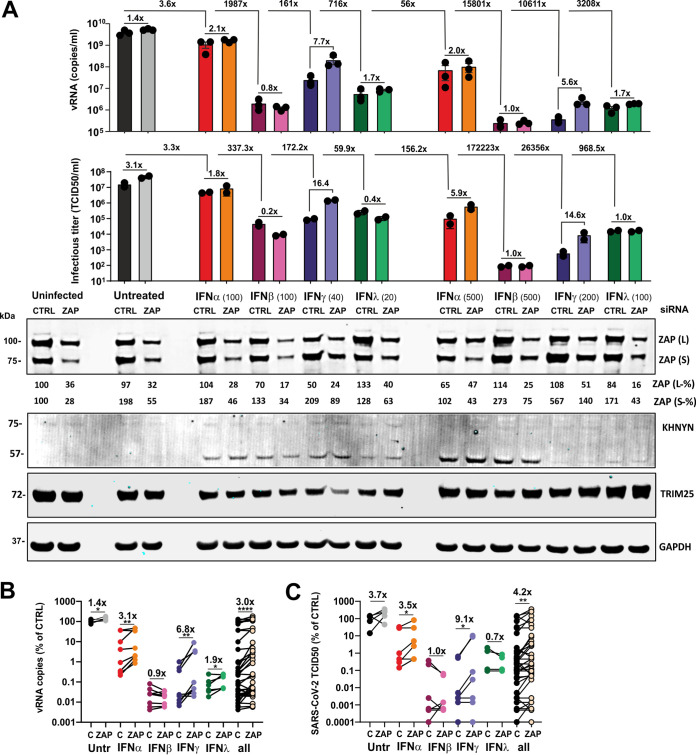
Role of ZAP in restricting SARS-CoV-2 production. (A) SARS-CoV-2 RNA levels (top panel, *n* = 3 [biological replicates] ± SEM) and infectious titers (middle panel, *n* = 2 [biological replicates] ± standard deviations [SD]) in the supernatant of Calu-3 cells that were left untreated and/or uninfected or infected with SARS-CoV-2 and treated with the indicated amounts of IFNs. Cells were additionally transfected either with control siRNA (CTRL) or ZAP siRNA (ZAP) as indicated. Immunoblots of whole-cell lysates were stained with anti-ZAP, anti-KHNYN, anti-TRIM25, and anti-GAPDH (bottom panel). Relative expression levels of the long (L) or short (S) isoforms of ZAP are indicated below the blots. One representative blot of 2 biological replicates is shown. (B and C) SARS-CoV-2 RNA copy numbers (B) or infectious virus titers (C), quantified relative to the control in the supernatant of Calu-3 cells that were left untreated or treated with the indicated IFNs and transfected with control (C) or ZAP (ZAP) siRNA. Numbers above the samples indicate the average change in viral RNA copy numbers (vRNA) or infectious titer. *P* values: *, <0.05; **, <0.01; ***, <0.001; ****, <0.0001; unpaired Student's *t* test.

To examine whether endogenous ZAP restricts SARS-CoV-2 and contributes to the antiviral effect of IFNs, we performed siRNA KD studies in Calu-3 cells and infected them. Western blot analyses showed that SARS-CoV-2 infection alone enhances expression of the short isoform of ZAP about 2-fold and that this induction was further enhanced by IFN-β and IFN-γ treatment (Fig. 4A; see also Fig. S4A and B). On average, treatment with ZAP siRNA reduced both ZAP(L) and ZAP(S) expression levels by ∼60% without affecting TRIM25 and KHNYN expression levels (Fig. 4A; see also Fig. S4A and B). In the initial experiment, siRNA-mediated KD of ZAP increased the levels of SARS-CoV-2 RNA determined by RT-qPCR (Fig. S4C) in the absence of IFN by ∼40% (Fig. S4A). IFN-α treatment reduced virus yield ∼337-fold, and knockdown of ZAP by ∼80% increased viral RNA (vRNA) levels in the culture supernatants by 6.5-fold (Fig. S4A). In agreement with the titration experiments (Fig. 3C), IFN-β and IFN-γ were more effective than IFN-α and reduced SARS-CoV-2 production by ∼4,000-fold. IFN-λ was not available for the initial experiment, and ZAP siRNA KD had no significant effect on virus yields upon treatment with IFN-β and IFN-γ.

10.1128/mBio.01930-20.4 FIG S4 Expression and anti-SARS-CoV-2 activity of ZAP. (A) SARS-CoV-2 RNA levels (top panel; *n* = 3 [biological replicates] ± SD) and infectious titers (middle panel; *n* = 3 [biological replicates] ± SD) in the supernatant of Calu-3 cells that were left uninfected or infected with SARS-CoV-2 and treated with the indicated amounts of IFNs. Cells were additionally transfected either with control or ZAP siRNA (CTRL and ZAP) as indicated. Immunoblots of whole-cell lysates were stained with anti-ZAP, anti-KHNYN, anti-TRIM25, and anti-GAPDH (bottom panel). Relative levels of expression of the long (L) or short (S) isoforms of ZAP are indicated below the blots. One representative blot of two biological replicates is shown. (B) ZAP(L) and ZAP(S) expression levels in Calu-3 cells upon infection and IFN treatment relative to expression levels in untreated cells (100%). (C) Standard curve and raw qRT-PCR threshold cycle (*C_T_*) values corresponding to the SARS-CoV-2 RNA copy numbers per ml shown in panel A. (D) Raw qRT-PCR *C_T_* values for the viral RNA levels shown in Fig. 4A. Panels B and C show mean values (± SD) from three replicates. Download FIG S4, TIF file, 1.1 MB. Copyright © 2020 Nchioua et al. 2020 Nchioua et al. This content is distributed under the terms of the Creative Commons Attribution 4.0 International license.

Saturating effects and almost complete inhibition of SARS-CoV-2 by other antiviral factors in the presence of IFN-β and IFN-γ might explain the lack of an effect of ZAP siRNA KD on virus yield. To further assess this, we repeated the ZAP siRNA KD experiment, including the use of 5-fold lower quantities of the different IFNs than had been used in the previous setting as well as the use of IFN-λ (Fig. 4A). The results confirmed that IFN-β, IFN-γ, and IFN-λ are substantially more effective against SARS-CoV-2 than IFN-α (Fig. 4A). Again, ZAP KD slightly increased SARS-CoV-2 RNA levels in culture supernatants in the absence of IFNs and frequently more efficiently in its presence. The effects of ZAP KD upon IFN-γ treatment were particularly pronounced at the low and high doses (i.e., resulted in 7.7-fold and 5.6-fold changes, respectively) (Fig. 4A). On average, under all conditions, ZAP KD increased SARS-CoV-2 RNA production by 3.0-fold (Fig. 4B, right). The enhancing effect seen in the absence of IFN was modest (1.4-fold) but significant and consistent (Fig. 4B, left). The effect of ZAP KD on vRNA yield was most pronounced in the presence of IFN-γ (6.8×) or IFN-α (3.1×) and was modest (1.9×) or absent upon treatment with IFN-λ or IFN-β, respectively (Fig. 4B).

**FIG 5:**
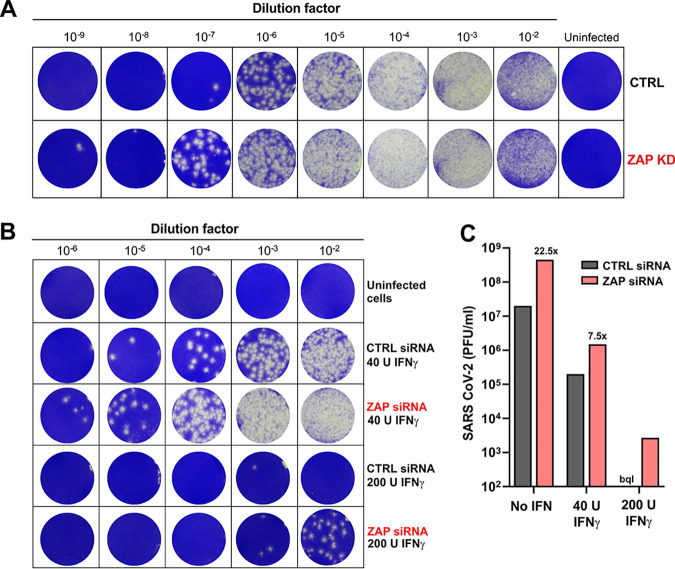
ZAP knockdown enhances infectious SARS-CoV-2 yield. (A and B) Cytopathic effects after infection of monolayers of Vero cells with serial dilutions of Calu-3 supernatants from the experiment represented in Fig. 4. Supernatants were obtained from Calu-3 cells treated with control or ZAP siRNA in the absence (A) or presence (B) of IFN-γ. Vero cells were stained with crystal violet (blue). (C) Calculated number of PFU in the supernatant of Calu-3 cells treated with control of ZAP siRNA. The number of plaques from cells treated with control siRNA and 200 U IFN-γ was too low for quantification (bql, below quantitation limit).

To further analyze the effects of IFN treatment and ZAP KD on SARS-CoV-2, we determined infectious virus yields in the culture supernatants. Results of 50% tissue culture infective dose (TCID_50_) endpoint titration correlated well with the RT-qPCR data (Fig. 4A; see also Fig. S3A; *R*^2^ = 0.713, *P* < 0.0001). On average, ZAP KD increased infectious virus yield 4.2-fold. In agreement with the vRNA data, the enhancing effect was most pronounced in the presence of IFN-γ (9.1×) and absent upon treatment with IFN-β or IFN-λ (Fig. 4C). The effects of ZAP KD on SARS-CoV-2 RNA yield and infectious titers were most obvious at nonsaturating levels of IFNs (Fig. 4B and C). To more exactly quantify the differences in infectious SARS-CoV-2 yields, we infected Vero cells with supernatants from the Calu-3 cells and determined virus-induced cytopathic effect (CPE) (Fig. 5). In the absence of IFN, knockdown (KD) of ZAP enhanced the number of plaque-forming units (PFU) per ml culture supernatant about 20-fold (Fig. 5A and C). Depletion of ZAP also clearly increased infectious SARS-CoV-2 yield in the presence of IFN-γ (Fig. 5B and C). For example, virus-induced plaques were absent at all dilutions in the presence of 200 U IFN-γ but were readily detectable at the 10^2^ and 10^3^ dilutions upon depletion of ZAP (Fig. 5B). Altogether, the results demonstrate that endogenous ZAP expression restricts SARS-CoV-2, especially in the presence of type II IFN.

### ZAP-L restricts SARS-CoV-2 more efficiently than ZAP-S.

**FIG 6:**
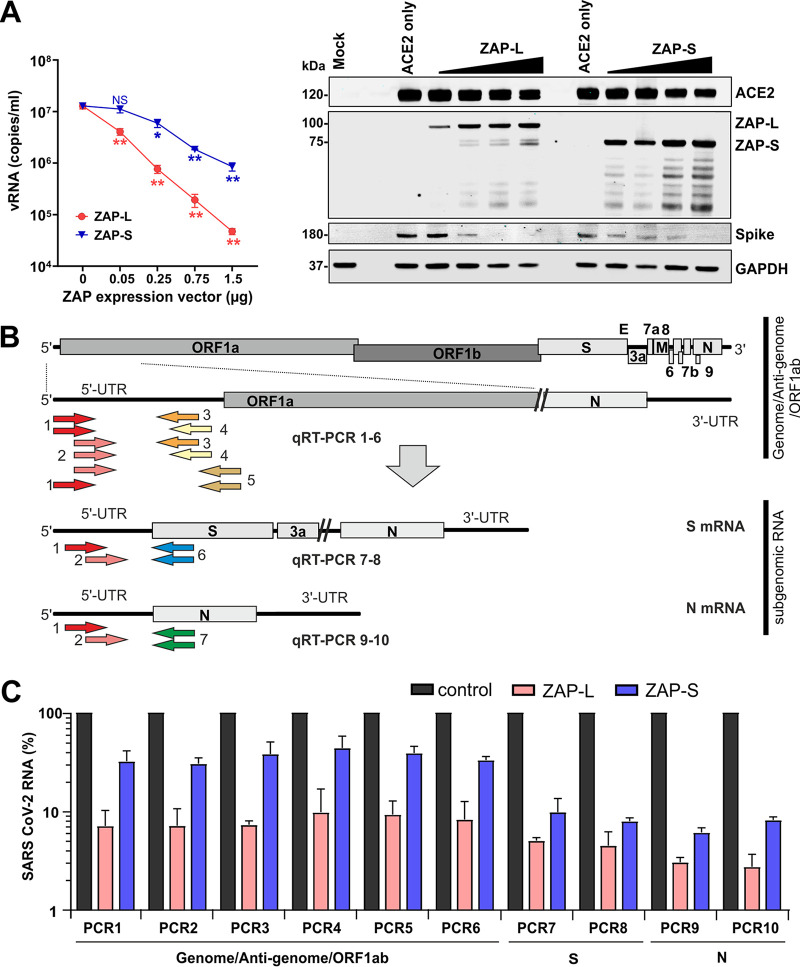
Restriction of SARS-CoV-2 by transient ZAP-L and ZAP-S expression. (A) Quantification of viral N gene RNA copies by qRT-PCR in the supernatant of ZAP KO HEK293T cells cotransfected with an ACE2 expression vector and increasing concentrations of vectors expressing ZAP-L or ZAP-S. Viral RNA yield was determined 48 h postinfection with SARS-CoV-2. Shown are mean values (±SD) obtained in three independent experiments, each measured in duplicate. Stars indicate significant (*P* value) differences from the no-ZAP control results as follows: *, <0.05; **, <0.01; ***, <0.001.The right panel shows a Western blot of whole-cell lysates stained with anti-ACE2, anti-ZAP-L and anti-ZAP-S, anti-SARS-CoV-2 Spike, and anti-GAPDH as loading control. (B) Schematic presentation of the SARS-CoV-2 genome and the positions of the primer binding sites in the full-length or subgenomic viral RNAs. (C) Effect of ZAP-L and ZAP-S on the levels of the indicated viral RNAs. Shown are mean values (±SD) from two independent experiments relative to the RNA levels obtained after cotransfection of ZAP-L or ZAP-S expression vectors compared to empty control vector (100%).

Human ZAP is expressed in two major isoforms containing identical N-terminal zinc-finger RNA binding motifs (38). The long isoform, named ZAP-L, contains an additional C-terminal catalytically inactive poly(ADP-ribose) polymerase (PARP) domain and S-farnesylation motif (38, 39). The precise role of both isoforms in the innate antiviral immune response remains to be determined, but previous reports proposed that ZAP-L is the major antiviral effector, while ZAP-S might play a role in immune modulatory activities (40–42). To determine their antiviral activities, we infected ZAP-KO HEK293T cells overexpressing ACE2 alone or together with different doses of ZAP-L or ZAP-S expression vectors and with SARS-CoV-2 and measured viral RNA yields in the culture supernatants by qPCR 2 days later. Overexpression of ZAP-L reduced viral RNA yields in a dose-dependent manner by up to 2 orders of magnitude or more (Fig. 6A). In comparison, ZAP-S had more modest effects but also achieved about 10-fold inhibition at the highest dose.

ZAP is known to restrict viral pathogens by promoting the degradation of CpG containing viral RNAs (10, 43, 44). To determine the effect of ZAP on the levels of SARS-CoV-2 RNAs, we performed qPCR assays with primer sets specific for the full-length RNAs (genome, antigenome, or ORF1ab mRNA) or with specific subgenomic mRNAs (Fig. 6B). Transcription of the coronavirus genome always starts at the 5′ UTR and after nucleotide 65 to 67 jumps and continues at the corresponding gene. All genes are subsequently transcribed in a consecutive manner until the 3′ UTR is reached. To detect specific mRNAs, we used primers binding to the beginning of the 5′ UTR present in all viral mRNA species and reverse primers binding to the beginning of the corresponding open reading frames (ORFs). We chose the beginning of the Spike (S) and nucleocapsid (N) ORFs because these represent the longest and shortest subgenomic mRNAs expressed by SARS-CoV-2, respectively (45). We used redundant sets of primers to assess possible biases resulting from the use of low binding primer combinations or from ineffective qPCR.

Our analyses revealed that coexpression of ZAP-L reduced the levels of full-length SARS-CoV-2 RNAs about 10-fold to 15-fold (Fig. 6C). In comparison, the levels of mRNAs encoding the S and N proteins were even reduced about 25-fold and 30-fold, respectively. Unexpectedly, ZAP-S reduced the levels of S-encoding and N-encoding viral mRNAs substantially (10-fold to 15-fold) more efficiently than those of the full-length SARS-CoV-2 RNAs (2-fold to 3-fold; Fig. 6C). Targeting of S-encoding mRNAs agrees with the reduction of S protein expression observed in the presence of ZAP (Fig. 6A). Notably, the strongest reduction was observed for the N gene (Fig. 6C), which showed about 2-fold-higher frequencies of CpGs than the average seen with the SARS-CoV-2 genome (Fig. 2D). Results were highly consistent for different primer sets, and control experiments verified that all PCRs yielded fragments of the expected size (data not shown). Altogether, these results confirmed that ZAP-L restricts SARS-CoV-2 more efficiently than ZAP-L and further revealed stronger effects on subgenomic than on full-length viral RNAs.

### The bat and pangolin orthologues of ZAP restrict SARS-CoV-2.

**FIG 7:**
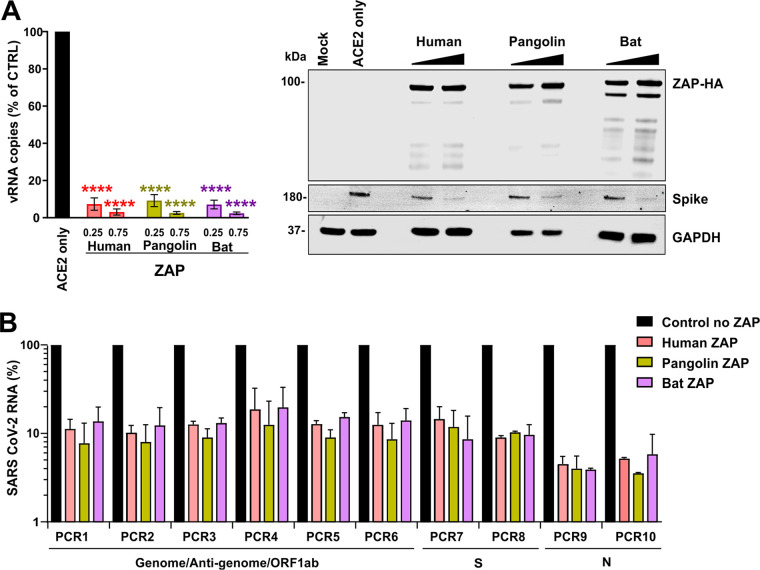
Restriction of SARS-CoV-2 by pangolin and horseshoe bat ZAP. (A) Viral N gene RNA copies detected in the supernatant of ZAP KO HEK293T cells cotransfected with an ACE2 expression vector and the indicated amounts of ZAP expression vectors compared to those transfected with the empty control vector (100%). Viral RNA yield was determined 48 h postinfection with SARS-CoV-2. Shown are mean values (±SD) obtained in three independent experiments, each measured in duplicate. Stars indicate significant differences from the no-ZAP control results as follows: *, <0.05; **, <0.01; ***, <0.001. The right panel shows a representative Western blot. (B) Effect of human, pangolin, and horseshoe bat ZAP expression on the levels of the indicated SARS-CoV-2 RNAs. Shown are mean values (±SD) from two independent experiments relative to the RNA levels obtained in the absence of ZAP (100%).

To determine whether the ability of ZAP to restrict SARS-CoV-2 is evolutionarily conserved, we performed infection experiments in ZAP-KO HEK293T cells expressing ACE2 alone or together with the human, pangolin, or horseshoe bat orthologues of ZAP. ZAP proteins derived from all three species were efficiently expressed and reduced the levels of SARS-CoV-2 RNA production in the cell culture supernatant by up to 2 orders of magnitude (Fig. 7A). The antiviral activity of ZAP orthologues from bat and pangolin did not differ significantly from human ZAP-L antiviral activity. qPCR analyses confirmed that all three ZAP orthologues reduced the levels of full-length SARS-CoV-2 mRNAs about 10-fold and the levels of S-encoding or N-encoding mRNAs about 12-fold or 30-fold, respectively (Fig. 7B). In agreement with the effects on viral mRNAs, all ZAP orthologues reduced the levels of S expression in SARS-CoV-2-infected cells (Fig. 7A). These results show that the anti-SARS-CoV-2 activity of ZAP is conserved in the reservoir bat and potential intermediate pangolin hosts.

## DISCUSSION

Coronaviruses generally show some degree of CpG suppression. In agreement with accumulating evidence (8, 22, 46), we show that the SARS-CoV-2 genome is particularly deficient in CpG dinucleotides and that the closest bat relatives of SARS-CoV-2 show the strongest suppression and lowest frequency of CpGs among all available bat CoV genomes. On average, CpGs are more strongly suppressed in humans than in bats, suggesting that the selection pressure for CpG suppression might be increased after zoonotic transmission. Consistent with this possibility, we found that coronaviruses associated with seasonal colds that are relatively well adapted to their human host showed significantly lower CpG frequencies and stronger suppression than their closest animal-infecting relatives. Bioinformatics-based studies proposed that efficient CpG suppression may allow SARS-CoV-2 to evade ZAP restriction but did not address this experimentally (8, 21). We show that knockdown of ZAP in viral target cells enhanced infectious SARS-CoV-2 production in both the presence and absence of IFN. However, we usually observed the strongest effects in the presence of IFN-γ, which is highly active against this viral pathogen. Altogether, our data demonstrate that ZAP efficiently restricts SARS-CoV-2 replication, although this virus shows relatively strong CpG suppression compared to other coronaviruses.

Consistent with increased selection pressure for CpG suppression in the human host, we found that community-acquired hCoVs showed lower frequencies of CpG dinucleotides than their closest animal relatives (Fig. 2A). This was not observed for highly pathogenic SARS-CoV and MERS-CoV, most likely reflecting less-advanced human adaptation consistent with their less-effective and transient spread. In addition, selection pressures may not act on all parts of the genome. Specifically, while SARS-CoV-2 shows low CpG frequencies throughout most parts of its genome, the number of CpGs at the 3′ end is higher. Notably, several ORFs overlap in this part of the genome, which might make it difficult for the virus to get rid of CpGs without a fitness cost. However, this might render SARS-CoV-2 vulnerable to ZAP restriction since many coronavirus mRNA transcripts encompass this region (45). Interestingly, it has been reported that almost all changes in the N gene, which contains higher numbers of CpGs (Fig. 2D), that have emerged during the spread of SARS-CoV-2 in the human population eliminate these dinucleotides, while this is less common in other parts of the genome (46). In agreement with efficient targeting, overexpression of ZAP reduced the levels of N-encoding mRNA more strongly than those of other SARS-CoV-2 RNA species (Fig. 6B; see also Fig. 7C). On average, the CpG frequency in the HIV-1 genome is substantially lower than in the SARS-CoV-2 genome. However, recently reported data show that a region of CpGs at the beginning of the *env* gene of HIV-1 rather than overall genomic content determines the susceptibility of HIV-1 to inhibition by ZAP (47). Thus, it is conceivable that parts of the SARS-CoV-2 genome may still be ZAP sensitive, although it shows strong CpG suppression throughout most parts of its genome. In fact, we found that ZAP-L efficiently reduced the levels of all three viral RNA species investigated, while ZAP-S reduced the levels of S-encoding and N-encoding mRNAs almost as efficiently as ZAP-L but had little effect on full-length SARS-CoV-2 RNAs (Fig. 6C). The molecular mechanisms underlying these differences warrant further investigation.

Results of further studies on the evolutionary constraints acting on SARS-CoV-2 and on further elimination of CpGs during human adaptation will be interesting. They might also reveal whether selection pressure for loss of CpGs might promote the emergence of less-virulent virus variants. For example, SARS-CoV-2 contains a unique potential furin cleavage site in its Spike protein that is absent in bat and pangolin viruses and that introduces several CpG dinucleotides (Fig. 2F). Increased furin-mediated activation of the Spike protein might affect viral infectivity as well as cell tropism and, consequently, its pathogenicity (48–50). It has been observed that this site may acquire mutations during viral passage in cell culture (51), and it will be of interest to determine whether this insertion increases ZAP sensitivity and whether it might also be prone to mutation or elimination *in vivo* (52).

SARS-CoV-2 is most closely related to two bat viruses (RaTG13 and RmYN02), which show about 96% sequence identity to the human virus (1, 18). However, the degrees of sequence homology are unevenly distributed throughout the viral genomes and it is under debate whether SARS-CoV-2 might represent a recombination between CoVs found in the reservoir bat host and the viruses found in intermediate hosts, such as pangolins, that also show high sequence identity to the human virus (20, 53). SARS-CoV-2 differs significantly from its bat relatives in the Spike coding region. Notably, we found that the RmYN02 bat CoV strain that is otherwise highly related to SARS-CoV-2 showed substantially higher CpG frequencies in the region encoding the ACE2 receptor binding region of the viral Spike (Fig. 2E). In this part of the genome, SARS-CoV-2 shows substantially higher similarity in sequence and CpG numbers to bat RaTG13 and pangolin Pang Guangdong CoV strains than to RmYN02 (20, 54). These differences and the possibility that recombination may have facilitated the loss of regions with high CpG frequencies warrant further investigation.

In agreement with recent data (5), we found that IFN-β efficiently inhibited SARS-CoV-2, while IFN-α was less effective. However, IFN-λ was about as effective as IFN-β. Most notably and in agreement with recent studies in colon organoids (55), type II IFN-γ showed the highest efficacy against SARS-CoV-2 (Fig. 3). More studies on the anti-SARS-CoV-2 activity of the various types of IFNs and their potential adverse effects in patients are required to optimize IFN-based therapeutic approaches. At least in Calu-3 cells, IFN-γ displayed the highest potency against SARS-CoV-2. IFN-γ has been used in a wide variety of clinical indications, and a tendency for higher levels of IFN-γ in moderate than in severe cases of COVID-19 has been reported (56–58). Thus, further studies on the application of IFN-γ in the treatment of COVID-19 are highly warranted.

Both SARS-CoV-2 infection alone and IFN treatment induced ZAP expression in our cell-based systems. This result agrees with the recent finding that ZAP mRNA expression is clearly induced in SARS-CoV-2-infected human individuals (37). In agreement with previous studies (38), the short isoform of ZAP in particular was induced by virus infection and IFN treatment, whereas the long isoform was constitutively expressed at relatively high levels but was minimally inducible. Knockdown of ZAP by ∼60% increased SARS-CoV-2 RNA yield by ∼40% in the absence of IFN. The enhancing effects of ZAP KD on SARS-CoV-2 RNA yield and infectious titers differed to some extent, due at least in part to variations in KD efficiencies and saturating effects in the presence of high levels of IFN-β, IFN-γ, and IFN-λ. In addition, the type of IFN seems to play a significant role. On average, KD of ZAP increased SARS-CoV-2 RNA and infectious virus yield by 6.8-fold and 9.1-fold upon treatment with IFN-γ but had no significant enhancing effect in the presence of IFN-β. In part, this can be explained by the fact that IFN-γ induced ZAP(S) expression in Calu-3 cells with higher efficiency than other IFNs. It will also be interesting to clarify whether IFN-β might be particularly effective in inducing other antiviral factors masking effects of ZAP. IFN treatment induces hundreds of potentially antiviral ISGs. Thus, it is remarkable that ZAP KD increased SARS-CoV-2 RNA yield and infectious titers ∼6-fold on average in the presence of IFN levels, resulting in robust but nonsaturating (i.e., 10-fold to 500-fold) inhibition of SARS-CoV-2. These results clearly show that ZAP restricts SARS-CoV-2 and contributes to the antiviral effect of IFNs. They further support the idea of a significant role of the short isoform of ZAP in restricting SARS-CoV-2 because it is more responsive to IFN treatment than the long isoform. It has been reported that it is mainly the long isoform of ZAP that exerts antiviral activity, while both suppressive and enhancing effects on the antiviral IFN response have been proposed for the short isoform (40–42). We found that ZAP-S is less effective in restricting SARS-CoV-2 than ZAP-L but also exerts significant inhibitory effects at higher expression levels (Fig. 6A) such as might be reached in the presence of IFNs (see Fig. S3 in the supplemental material). Altogether, the precise roles of the different isoforms of ZAP in innate antiviral immunity need further study and may depend on the viral pathogen as well as on the type of target cells.

In summary, our results show that ZAP restricts SARS-CoV-2 despite relatively strong CpG suppression. Our data confirm that SARS-CoV-2 is highly susceptible to IFNs and might motivate assessment of combination therapies that include IFN-γ for treatment of COVID-19. If SARS-CoV-2 is highly sensitive to IFN, why is it frequently not efficiently controlled by the innate immune response? It was known previously that coronaviruses use various mechanisms to avoid immune sensing (35, 59, 60), and recent data show that SARS-CoV-2 Nsp1 proteins block ribosomal translation of cellular proteins, including antiviral defense factors (61). However, we are still far from a full understanding of viral immune evasion and counteraction mechanisms. ZAP is only one of numerous effectors of the antiviral immune response. Many others remain to be defined, and it will be important to determine whether the different types of IFN use different effectors to restrict SARS-CoV-2. A better knowledge of the anti-SARS-CoV-2 effectors of the IFN response and the viral countermeasures will help to develop safe and effective immune therapy approaches against COVID-19.

## MATERIALS AND METHODS

### Phylogenetic analyses.

Nucleotide sequences of full-length or nearly full-length coronavirus genomes were obtained from the NCBI or GISAID (62) database and aligned using Clustal Omega (https://www.ebi.ac.uk/Tools/msa/clustalo/) (63). Phylogenetic trees showing distance-based relationship inference data based on representative full-genome nucleotide sequences were generated using the NGPhylogeny.fr FastME tool (https://ngphylogeny.fr/) (64) and were visualized using iTOL (https://itol.embl.de/) (65).

### Cell culture.

Vero E6 cells (Cercopithecus aethiops-derived epithelial kidney cells, ATCC) were grown in Dulbecco’s modified Eagle’s medium (DMEM; Gibco, catalog no. 41965039) supplemented with 2.5% heat-inactivated fetal calf serum (FCS; Gibco, catalog no. 10270106), 100 units/ml penicillin, 100 μg/ml streptomycin (Thermo Fisher, catalog no. 15140122), 2 mM l-glutamine, 1 mM sodium pyruvate (Pan Biotech, catalog no. P04-8010), and 1× nonessential amino acids (Sigma, catalog no. M7145). Caco-2 cells (human epithelial colorectal adenocarcinoma cells, kindly provided by H. Barth, Ulm University) were grown in the same media but with supplementation of 10% FCS. Calu-3 cells (human epithelial lung adenocarcinoma cells, kindly provided by M. Frick, Ulm University) were cultured in minimum essential Eagle medium (MEM; Sigma, catalog no. M4655) supplemented with 10% FCS (during viral infection) or 20% FCS (at all other times), 100 units/ml penicillin, 100 μg/ml streptomycin, 1 mM sodium pyruvate, and 1× nonessential amino acids. NHLF cells (primary human lung fibroblasts; Lonza), HEK293T ZAP KO cells (10), and A549 cells (adenocarcinoma human alveolar basal epithelial cells; ATCC) were cultured in DMEM supplemented with 10% FCS, 2 mM μg/ml l-glutamine, 100 units/ml penicillin, and 100 μg/ml streptomycin.

### Dinucleotide content and CpG sliding window analysis.

Frequencies of dinucleotides (CpG or UpA) were calculated as the number of dinucleotides present in the viral gene or genome divided by its base pair length, whereas dinucleotide suppression was calculated as (number of dinucleotide XpY * mRNA sequence length)/(number of X nucleotides * number of Y nucleotides). Representative full-genome sequences of coronaviruses (NCBI accession numbers are listed in Table S1 in the supplemental material) were aligned using DECIPHER with a gap opening/extension penalty of 70, to avoid formation of large gaps. Numbers of CpGs were extracted using a sliding window of 100 nucleotides and a step size of 100. All analyses were done using R. The raw data are displayed as a heat map generated by GraphPad Prism.

### Species-specific CpG content.

mRNA sequences were extracted from genome transcripts (NCBI) of Homo sapiens (assembly GRCh38.p13), Rhinolophus ferrumequinum (assembly mRhiFer1_v1.p), Manis javanica (assembly ManJav1.0), Camelus dromedarius (assembly CamDro3), Bos taurus (assembly ARS-UCD1.2), Sus scrofa (assembly Sscrofa11.1), and Rattus rattus (assembly Rrattus_CSIRO_v1). CpG suppression was calculated as (number of CpG * mRNA sequence length)/(number of C * number of G).

### Quantitative real-time PCR (qRT-PCR).

N (nucleoprotein) transcript levels were determined in supernatants collected from SARS-CoV-2-infected Calu-3 cells 48 h postinfection. Total RNA was isolated using a viral RNA minikit (Qiagen, catalog no. 52906) according to the manufacturer’s instructions. RNA concentrations were determined using a NanoDrop 2000 spectrophotometer. Real-time quantitative PCR (RT-qPCR) was performed as previously described (66) using TaqMan Fast Virus 1-step master mix (Thermo Fisher, catalog no. 4444436) and a OneStepPlus real-time PCR system (96-well format, fast mode). Primers were purchased from Biomers (Ulm, Germany) and dissolved in nuclease-free water. Synthetic SARS-CoV-2 RNA (Twist Bioscience, catalog no. 102024) or RNA isolated from BetaCoV/France/IDF0372/2020 viral stocks quantified via the use of this synthetic RNA (for low-threshold-cycle [*C_T_*] samples) was used as a quantitative standard to obtain viral copy numbers. All reactions were run in duplicate. Sequences of the primers and probe were as follows: forward primer (HKU-NF), 5′-TAA TCA GAC AAG GAA CTG ATT A-3′; reverse primer (HKU-NR), 5′-CGA AGG TGT GAC TTC CAT G-3′; probe (HKU-NP), 5′-FAM (6-carboxyfluorescein)-GCA AAT TGT GCA ATT TGC GG-TAMRA (6-carboxytetramethylrhodamine)-3′.

### SYBR green qRT-PCRs.

Total RNA was isolated using a viral RNA minikit (Qiagen, catalog no. 52906) according to the manufacturer’s instructions. Residual genomic DNA was removed from RNA preparations using a DNA-free DNA removal kit (catalog no. AM1906; Thermo Fisher). RNA concentrations were determined using a NanoDrop 2000 spectrophotometer, and for each sample, equal RNA amounts were subjected to cDNA synthesis using a PrimeScript reverse transcription reagent kit (catalog no. RR037A; TaKaRa) with random 6-mers and oligo(dT) primers. Reactions without reverse transcriptase were included as controls to exclude contamination with genomic DNA. cDNA was used for SYBR green PCRs using PowerUp SYBR green master mix (catalog no. A25777; Thermo Fisher), and viral primer sets were used in reactions normalized for RPL4 (ribosomal protein L4) mRNA levels as an internal control. SARS-CoV-2 primers were designed as follows: primer 1, 5′-ATA CCT TCC CAG GTA ACA AAC CA-3′; primer 2, 5′-CCA ACC AAC TTT CGA TCT CTT GTA GA-3′; primer 3, 5′-GTC CTG TCA ACG ACA GTA ATT AGT TAT TAA TTA TAC-3′; primer 4, 5′-AAA CCT AGA TGT GCT GAT GAT CG-3′; primer 5, 5′-CTC CAT CTT ACC TTT CGG TCA CA-3′; primer 6, 5′-GAA AAA CAA ACA TTG TTC GTT TAG TTG TTA AC-3′; primer 7, 5′-CAT TAT CAG ACA TTT TAG TTT GTT CGT TTA GAT G-3′. RPL4 primers were as follows: forward, 5′-ACG ATA CGC CAT CTG TTC TGC C-3′; and reverse, 5′-GGA GCA AAA CAG CTT CCT TGG TC-3′. The specificity of the different primers was assessed by testing the respective primer sets on infected and noninfected samples.

### Expression constructs.

Vectors encoding human ZAP-L and ZAP-S were generated by cloning human ZAP (GenScript ORF cDNA clone OHu25350) into the pCG internal ribosome entry site (IRES) blue fluorescent protein (BFP) expression construct via XbaI/MluI sites. Vectors encoding ZAP-L and ZAP-XL containing an N-terminal hemagglutinin (HA) tag from the Sunda pangolin (Manis javanica) and the Greater horseshoe bat (Rhinolophus ferrumequinum), respectively, were synthetized by BaseClear and subcloned into pCG IRES BFP expression plasmid via XbaI/MluI sites. The sequence of the insertion was confirmed to match NCBI reference sequences (accession no. XM_017681059.1 and XM_033098708.1). ACE2 expression plasmid was kindly provided by Kei Sato.

### Transfection and infection of HEK293T ZAP KO cells.

A total of 0.4 million HEK293T ZAP KO cells were cotransfected using polyethylenimine (PEI) transfection reagent with 0.5 μg of the ACE2 expression construct and indicated concentrations of pCG HA-ZAP IRES BFP from human (ZAP L), Sunda pangolin (Manis javanica) (ZAP L), or Greater horseshoe bat (Rhinolophus ferrumequinum) (ZAP XL) or of pCG ZAP IRES BFP (L or S) expression vectors. Total DNA amount was normalized to 2.5 μg by adding control pCG IRES BFP vector. At 24 h posttransfection, the medium was changed and the transfected cells were infected with SARS-CoV-2 (multiplicity of infection [MOI] 0.05). At 6 h postinfection, the medium was changed and the cells were supplemented with fresh media. At 48 h postinfection, the cells and supernatants were harvested for further analysis.

### SDS-PAGE and Western blotting.

SDS-PAGE and Western blotting were performed as previously described (45). In brief, at 2 days postinfection, cells were washed with phosphate-buffered saline (PBS) and lysed with coimmunoprecipitation (Co-IP) buffer. The samples were separated on 4% to 12% Bis-Tris gradient acrylamide gels (Invitrogen) and blotted onto polyvinylidene difluoride (PVDF). Blotted membranes were probed with anti-ZAP (GeneTex, catalog no. GTX120134) diluted 1 to 1,000 or with anti-HA tag (catalog no. C29F4; Cell Signaling) diluted 1 to 2,000, anti-KHNYN (Santa Cruz Biotechnology, catalog no. sc-514168) diluted 1 to 50, or anti-TRIM25 (BD Biosciences, catalog no. 610570) or with anti-GAPDH (BioLegend, catalog no. 607902) or anti-beta-actin (abcam, catalog no. ab8227), each diluted 1 to 2,000, or anti-ISG15 (Santa Cruz Biotechnology, catalog no. sc-166755) diluted 1 to 1,000. Proteins were also stained using anti-ACE2 (abcam, catalog no. ab15348) and anti-SARS-CoV-2 Spike (Biozol, catalog no. GTX-GTX632604) primary antibodies, both diluted to 1 to 1,000. Subsequently, blots were stained with IRDye 680RD goat anti-rabbit IgG (H+L) (Li-Cor, catalog no. 926-68071), IRDye 800CW goat anti-mouse IgG (H+L), and IRDye 800CW goat anti-rat IgG (H+L) (Li-Cor, catalog no. 925-32219) secondary antibodies, all diluted 1 to 20,000, and were scanned using a Li-Cor Odyssey reader.

### Effect of IFNs on SARS-CoV-2 replication.

A total of 300,000 Calu-3 cells were seeded in 12-well plates. At 24 h and 96 h postseeding, cells were stimulated with increasing amounts of IFNs (IFN-α2, IFN-β, and IFN-γ; 0.8, 4, 20, 100, and 500 U/ml) or of IFN-λ1 (0.16, 0.8, 4, 20, and 100 ng) per ml of MEM medium. At 24 h after the first stimulation, the medium was exchanged. At 2 h after the second stimulation, Calu-3 cells were infected with SARS-CoV-2 (MOI 0.05), and 7 to 9 h later, supernatant was removed and 1 ml fresh medium was added. At 48 h postinfection, cells were harvested for further analysis.

### ZAP knockdown and IFN treatment in Calu-3 cells.

A total of 300,000 Calu-3 cells were seeded in 12-well plates. At 24 h and 96 h postseeding, they were transfected either with a nontargeting control siRNA (Eurofins, UUC UCC GAA CGU GUC ACG UdT dT) or with ZAP-specific siRNA (siRNA SMARTpool; Dharmacon, catalog no. SO-2863397G) (10). siRNA (20 μM) was transfected in one well using Lipofectamine RNAiMAX (Thermo Fisher) according to the manufacturer’s instructions. Prior to transfection, the medium was changed. At 14 h posttransfection, the medium was replaced with 1 ml MEM supplemented with IFNs (100 or 500 U/ml IFN-α2, 100 or 500 U/ml IFN-β, 40 or 200 U/ml IFN-γ, and 20 or 100 ng IFN-λ1). At 7 h posttransfection, the Calu-3 cells were infected with SARS-CoV-2 (MOI 0.05), and 7 to 9 h later, the supernatant was removed and 1 ml fresh medium was added. At 48 h postinfection, cells and supernatants were harvested for further analysis.

### Virus strains and propagation.

BetaCoV/Netherlands/01/NL/2020 or BetaCoV/France/IDF0372/2020 was obtained from the European Virus Archive. The virus was propagated by infecting 70% confluent Vero E6 in 75-cm^2^ cell culture flasks at an MOI of 0.003 in 3.5 ml serum-free medium containing 1 μg/ml trypsin. The cells were incubated for 2 h at 37°C followed by addition of 20 ml medium containing 15 mM HEPES. The DMEM was changed after 3 days postinfection and the supernatant harvested 48 h postinfection upon the observation of visible cytopathic effect (CPE). Supernatants were centrifuged for 5 min at 1,000 × *g* to remove debris, aliquoted, and stored at −80°C. Infectious virus titer was determined as PFU or TCID_50_. Genomic RNA copies were determined by RT-qPCR.

### PFU assay.

To determine PFU counts, SARS-CoV-2 stocks were serially diluted 10-fold. Monolayers of Vero E6 cells in 12 wells were infected with the dilutions and incubated for 1 to 3 h at 37°C with shaking every 15 to 30 min. Afterward, the cells were overlaid with 1.5 ml of 0.8% Avicel RC-581 (FMC Corporation) in medium and incubated for 3 days. Cells were fixed by adding 1 ml 8% paraformaldehyde (PFA) and incubated at room temperature for 45 min. After the supernatant had been discarded, the cells were washed once with PBS, and 0.5 ml of staining solution (0.5% crystal violet–0.1% Triton–water) was added. After 20 min incubation at room temperature, the staining solution was removed using water, virus-induced plaque formation quantified, and PFU per ml calculated.

### TCID_50_ endpoint titration.

SARS-CoV-2 stocks or infectious supernatants were serially diluted. A total of 25,000 Caco-2 cells were seeded per well in 96-well flat-bottom plates in 100 μl medium and incubated overnight followed by addition of 62 μl fresh medium. Next, 18-μl volumes of titrated SARS-CoV-2 stocks or supernatant were used for infection, resulting in final dilutions of 1:10^1^ to 1:10^9^ on the cells in triplicate. Cells were then incubated for 6 days and monitored for CPE. TCID_50_/ml was calculated according to the method of Reed and Muench (67).

### Calu-3, A549, and NHLF stimulation.

Calu-3, A549, and NHLF cells were seeded in 12-well plates and stimulated with interleukin-2 (IL-2) (10 ng/ml), phytohemagglutinin (PHA) (25 ng/ml), IL-27 (5 ng/ml), tumor necrosis factor alpha (TNF-α) (25 ng/ml), IFN-α2 (500 U/ml), IFN-β (500 U/ml), IFN-γ (200 U/ml), and IFN-λ1 (100 ng). At 3 and 6 days poststimulation, cells were harvested and lysed for Western blot analysis. Calu-3 cells were stimulated for 24 h only due to cytopathic effects of the IFN treatment.

### Statistical analysis.

Statistical analyses were performed with GraphPad Prism (GraphPad Software) and Microsoft Excel. *P* values were calculated using the two-tailed unpaired Student's *t* test unless specified otherwise. Correlations were calculated with the linear regression module. Unless stated otherwise, all experiments were performed in duplicate. Significant differences are indicated as follows: *, *P* < 0.05; **, *P* < 0.01; ***, *P* < 0.001. Statistical parameters are specified in the figure legends.
