# Supplementary material for: SARS-CoV-2 Is Restricted by Zinc Finger Antiviral Protein despite Preadaptation to the Low-CpG Environment in Humans
Source: mBio. 2020 Oct 16;11(5):e01930-20. doi: 10.1128/mBio.01930-20 (PMC7569149; doi:10.1128/mBio.01930-20)
Supplement: TABLE S1 [file mBio.01930-20-st001.docx]

**Supplementary Table 1.** Features of Coronavirus sequences analyzed.

| **Viral isolate** | seq length | no.  of CpG | no.  of UpA | CpG freq. | UpA freq. | CpG suppr. | UpA suppr. | GC content % | % identity to ref. | accession no./  ID |
| --- | --- | --- | --- | --- | --- | --- | --- | --- | --- | --- |
| **SARS-hCoV-2 Wuhan-Hu-1** | **29872** | **436** | **2375** | **0.0146** | **0.0795** | **0.406** | **0.827** | **38.0** |  | **NC_045512.2** |
| SARS-hCoV-2 TWN/CGMH-CGU-01/2020 | 29862 | 439 | 2373 | 0.0147 | 0.0795 | 0.408 | 0.828 | 38.0 | 99 | MT192759.1 |
| SARS-hCoV-2 USA/MN3-MDH3/2020 | 29783 | 439 | 2368 | 0.0147 | 0.0795 | 0.409 | 0.829 | 38.0 | 99 | MT188339.1 |
| SARS-hCoV-2 BRA/SP02cc/2020 | 29903 | 440 | 2378 | 0.0147 | 0.0795 | 0.409 | 0.828 | 38.0 | 99 | MT350282.1 |
| bat CoV RaTG13 | 29855 | 441 | 2384 | 0.0148 | 0.0799 | 0.409 | 0.833 | 38.0 | 96 | MN996532.1 |
| bat SARS RmYN02 | 29671 | 451 | 2393 | 0.0152 | **0.0807** | 0.416 | 0.847 | 38.2 | 93 | EPI_ISL_412977 |
| pangolin CoV PCoV_GX-P2V | 29795 | 461 | 2300 | 0.0155 | 0.0772 | 0.418 | 0.818 | 38.5 | 85 | MT072864.1 |
| pangolin CoV PCoV_GX-P1E | 29801 | 459 | 2295 | 0.0154 | 0.0770 | 0.416 | 0.815 | 38.5 | 85 | MT040334.1 |
| pangolin CoV-19/pangolin/Guangdong/1/2019 | 29825 | 428 | 2348 | 0.0144 | 0.0787 | 0.393 | 0.826 | 38.2 | 90 | EPI_ISL_410721 |
|  | | | | | | | | | | |
| **SARS hCoV Tor2** | **29751** | **568** | **2081** | **0.0191** | **0.0699** | **0.460** | **0.798** | **40.8** |  | **NC_004718.3** |
| SARS hCoV GD01 | 29757 | 570 | 2074 | 0.0192 | 0.0697 | 0.460 | 0.797 | 40.8 | 99 | AY278489.2 |
| SARS hCoV CUHK-W1 | 29736 | 567 | 2075 | 0.0191 | 0.0698 | 0.459 | 0.797 | 40.8 | 99 | AY278554.2 |
| SARS hCoV ZS-C | 29647 | 566 | 2065 | 0.0191 | 0.0697 | 0.460 | 0.795 | 40.8 | 99 | AY395003.1 |
| civet SARS CoV SZ16 | 29731 | 569 | 2080 | 0.0191 | 0.0700 | 0.459 | 0.800 | 40.8 | 99 | AY304488.1 |
| civet SARS CoV civet007 | 29540 | 569 | 2058 | 0.0193 | 0.0697 | 0.462 | 0.797 | 40.8 | 99 | AY572034.1 |
| civet SARS CoV SZ3 | 29741 | 569 | 2078 | 0.0191 | 0.0699 | 0.459 | 0.799 | 40.8 | 99 | AY304486.1 |
| civet SARS CoV civet010 | 29518 | 568 | 2057 | 0.0192 | 0.0697 | 0.461 | 0.798 | 40.8 | 99 | AY572035.1 |
| bat CoV WIV16 | 30290 | 596 | 2117 | 0.0197 | 0.0699 | 0.471 | 0.802 | 40.9 | 94 | KT444582.1 |
| bat CoV BtRs-BetaCoV/YN2018A | 29698 | 596 | 2043 | 0.0201 | 0.0688 | 0.477 | 0.793 | 41.0 | 93 | MK211375.1 |
| bat CoV BtRs-BetaCoV/YN2018B | 30256 | 588 | 2106 | 0.0194 | 0.0696 | 0.466 | 0.797 | 40.8 | 94 | MK211376.1 |
| bat CoV BtRs-BetaCoV/YN2018C | 29689 | 589 | 2006 | 0.0198 | 0.0676 | 0.470 | 0.780 | 41.1 | 94 | MK211377.1 |
| bat CoV Rs4874 | 30311 | 597 | 2116 | 0.0197 | 0.0698 | 0.471 | 0.800 | 40.9 | 94 | KY417150.1 |
|  | | | | | | | | | | |
| **MERS hCoV-EMC** | **30119** | **711** | **2302** | **0.0236** | **0.0764** | **0.555** | **0.896** | **41.2** |  | **NC_019843.3** |
| MERS hCoV Al-Hasa_1_2013 | 30117 | 713 | 2309 | 0.0237 | 0.0767 | 0.558 | 0.897 | 41.2 | 99 | KF186567.1 |
| MERS hCoV 2c England-Qatar/2012 | 30112 | 714 | 2306 | 0.0237 | 0.0766 | 0.559 | 0.896 | 41.2 | 99 | KC667074.1 |
| MERS hCoV KNIH/002_05_2015 | 30108 | 711 | 2312 | 0.0236 | 0.0768 | 0.558 | 0.897 | 41.1 | 99 | MK796425.1 |
| camel MERS CoV Egypt/Camel/AHRI-FAO-1/2018 | 30106 | 722 | 2313 | 0.0240 | 0.0768 | 0.566 | 0.899 | 41.2 | 99 | MK967708.1 |
| camel MERS CoV UAE_B73_2015 | 30123 | 716 | 2308 | 0.0238 | 0.0766 | 0.561 | 0.896 | 41.2 | 99 | MF598663.1 |
| camel MERS CoV Qatar_2_2014 | 30117 | 713 | 2306 | 0.0237 | 0.0766 | 0.559 | 0.895 | 41.2 | 99 | KJ650098.1 |
| camel MERS CoV UAE_B39_2015 | 30123 | 711 | 2305 | 0.0236 | 0.0765 | 0.560 | 0.896 | 41.1 | 99 | MF598631.1 |
| bat CoV PREDICT/PDF-2180 | 29642 | 659 | 2283 | 0.0222 | 0.0770 | 0.525 | 0.903 | 41.2 | 82 | NC_034440.1 |
| bat CoV Neoromicia/PML-PHE1/RSA/2011 | 30111 | 626 | 2439 | 0.0208 | 0.0810 | 0.517 | 0.915 | 40.1 | 85 | KC869678.4 |
|  | | | | | | | | | | |
| **OC43 hCoV ATCC VR-759** | **30741** | **485** | **2794** | **0.0158** | **0.0909** | **0.481** | **0.924** | **36.8** |  | **NC_006213.1** |
| OC43 hCoV 1908A/2010 | 30719 | 464 | 2787 | 0.0151 | 0.0907 | 0.462 | 0.921 | 36.7 | 99 | KF923886.1 |
| OC43 hCoV HK04-01 | 30710 | 471 | 2789 | 0.0153 | 0.0908 | 0.469 | 0.922 | 36.7 | 99 | JN129834.1 |
| OC43 hCoV HK04-02 | 30722 | 469 | 2793 | 0.0153 | 0.0909 | 0.468 | 0.921 | 36.7 | 99 | JN129835.1 |
| OC43 hCoV MY-U1024/12 | 30716 | 463 | 2797 | 0.0151 | 0.0911 | 0.463 | 0.922 | 36.6 | 99 | KX538975 |
| OC43 hCoV GZYF-26 | 30530 | 457 | 2787 | 0.0150 | 0.0913 | 0.460 | 0.924 | 36.6 | 98 | MG197715 |
| bovine CoV Kakegawa | 31038 | 485 | 2832 | 0.0156 | 0.0912 | 0.471 | 0.937 | 37.0 | 96 | AB354579.1 |
| bovine CoV BCoV-ENT | 31028 | 498 | 2821 | 0.0161 | 0.0909 | 0.481 | 0.936 | 37.1 | 95 | NC_003045.1 |
| bovine CoV Mebus | 31032 | 484 | 2834 | 0.0156 | 0.0913 | 0.470 | 0.937 | 37.0 | 96 | U00735.2 |
| bovine CoV DB2 | 31007 | 486 | 2811 | 0.0157 | 0.0907 | 0.469 | 0.934 | 37.1 | 96 | DQ811784.2 |
| porcine CoV PHEV CC14 | 30682 | 504 | 2738 | 0.0164 | 0.0892 | 0.486 | 0.923 | 37.3 | 93 | MF083115.1 |
| porcine CoV PHEV USA-15TOSU1362 | 30515 | 506 | 2737 | 0.0166 | 0.0897 | 0.495 | 0.924 | 37.1 | 92 | KY419110.1 |
| porcine CoV PHEV VW572 | 30480 | 504 | 2748 | 0.0165 | 0.0902 | 0.490 | 0.931 | 37.2 | 93 | DQ011855.1 |
|  | | | | | | | | | | |
| **HKU1 hCoV** | **29926** | **340** | **3205** | **0.0114** | **0.1071** | **0.458** | **0.959** | **32.1** |  | **NC_006577.2** |
| HKU1 hCoV N25 genotype B | 29845 | 324 | 3228 | 0.0109 | 0.1082 | 0.440 | 0.969 | 32.0 | 95 | DQ415902.1 |
| HKU1 hCoV N22 genotype C | 29905 | 346 | 3242 | 0.0116 | 0.1084 | 0.468 | 0.970 | 32.0 | 96 | DQ415899.1 |
| HKU1 hCoV BJ01-p3 | 29887 | 338 | 3214 | 0.0113 | 0.1075 | 0.459 | 0.961 | 32.0 | 99 | KT779555.1 |
| rodent CoV RtNn-CoV/SAX2015 | 31172 | 585 | 2666 | 0.0188 | 0.0855 | 0.523 | 0.919 | 38.4 | 74 | KY370049.1 |
| rodent CoV VZ_BetaCoV_16715_52 | 31083 | 572 | 2750 | 0.0184 | 0.0885 | 0.530 | 0.935 | 37.8 | 74 | MH687968.1 |
| rodent CoV RtBi-CoV/FJ2015 | 31149 | 601 | 2636 | 0.0193 | 0.0846 | 0.533 | 0.915 | 38.6 | 74 | KY370051.1 |
|  | | | | | | | | | | |
| **229E hCoV** | **27317** | **488** | **2034** | **0.0179** | **0.0745** | **0.496** | **0.793** | **38.3** |  | **NC_002645.1** |
| 229E hCoV 229E/Seattle/USA/SC399/2016 | 27055 | 473 | 2036 | 0.0175 | 0.0753 | 0.492 | 0.799 | 38.0 | 97 | KY674914.1 |
| 229E hCoV 229E/Haiti-1/2016 | 27271 | 478 | 2059 | 0.0175 | 0.0755 | 0.494 | 0.798 | 38.0 | 98 | MF542265.1 |
| 229E hCoV 229E/BN1/GER/2015 | 27022 | 473 | 2030 | 0.0175 | 0.0751 | 0.490 | 0.796 | 38.1 | 97 | KU291448.1 |
| camel CoV camel/Jeddah/N60/2014 | 27390 | 497 | 2076 | 0.0181 | 0.0758 | 0.499 | 0.812 | 38.4 | 92 | KT368899.1 |
| camel CoV camel/Taif/T96/2015 | 27392 | 498 | 2077 | 0.0182 | 0.0758 | 0.501 | 0.812 | 38.4 | 92 | KT368914.1 |
| camel CoV camel229E-CoV/JC49/KSA/2014 | 27193 | 490 | 2058 | 0.0180 | 0.0757 | 0.496 | 0.811 | 38.4 | 91 | KT253325.1 |
| bat CoV BtCoV/KW2E-F151/Hip_cf._rub/GHA/2011 | 28026 | 519 | 2100 | 0.0185 | 0.0749 | 0.497 | 0.814 | 38.9 | 88 | KT253269.1 |
| bat CoV BtKY229E-8 | 27636 | 522 | 2050 | 0.0189 | 0.0742 | 0.502 | 0.809 | 39.1 | 85 | KY073748.1 |
|  | | | | | | | | | | |
| **NL63 hCoV Amsterdam I** | **27553** | **332** | **2493** | **0.0120** | **0.0905** | **0.417** | **0.876** | **34.5** |  | **NC_005831.2** |
| NL63 hCoV NL63/Seattle/USA/SC0768/2019 | 27537 | 327 | 2461 | 0.0119 | 0.0894 | 0.419 | 0.886 | 34.1 | 98 | MN306040.1 |
| NL63 hCoV Amsterdam 057 | 27550 | 329 | 2488 | 0.0119 | 0.0903 | 0.412 | 0.876 | 34.5 | 99 | DQ445911.1 |
| NL63 hCoV Kilifi_HH_5402_20-May-2010 | 27832 | 327 | 2506 | 0.0117 | 0.0900 | 0.406 | 0.872 | 34.5 | 98 | MG428704.1 |
| bat CoV BtKYNL63-9a | 28363 | 609 | 2003 | 0.0215 | 0.0706 | 0.563 | 0.782 | 39.2 | 76 | KY073744.1 |
