# Supplementary figures and images for: SARS-CoV-2 Is Restricted by Zinc Finger Antiviral Protein despite Preadaptation to the Low-CpG Environment in Humans
Source: mBio. 2020 Oct 16;11(5):e01930-20. doi: 10.1128/mBio.01930-20 (PMC7569149; doi:10.1128/mBio.01930-20)

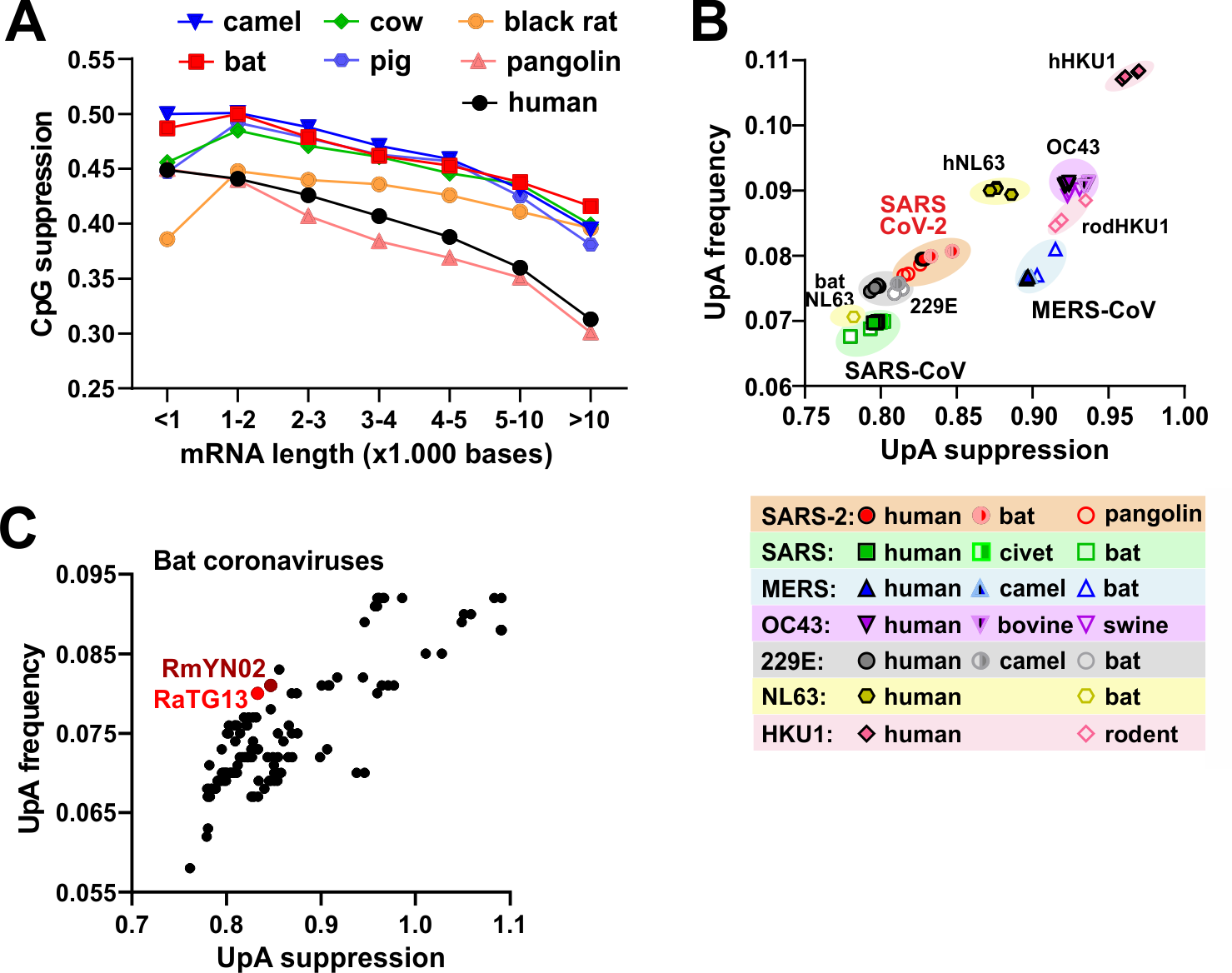

Supplement: FIG S1 [file mBio.01930-20-sf001.tif]

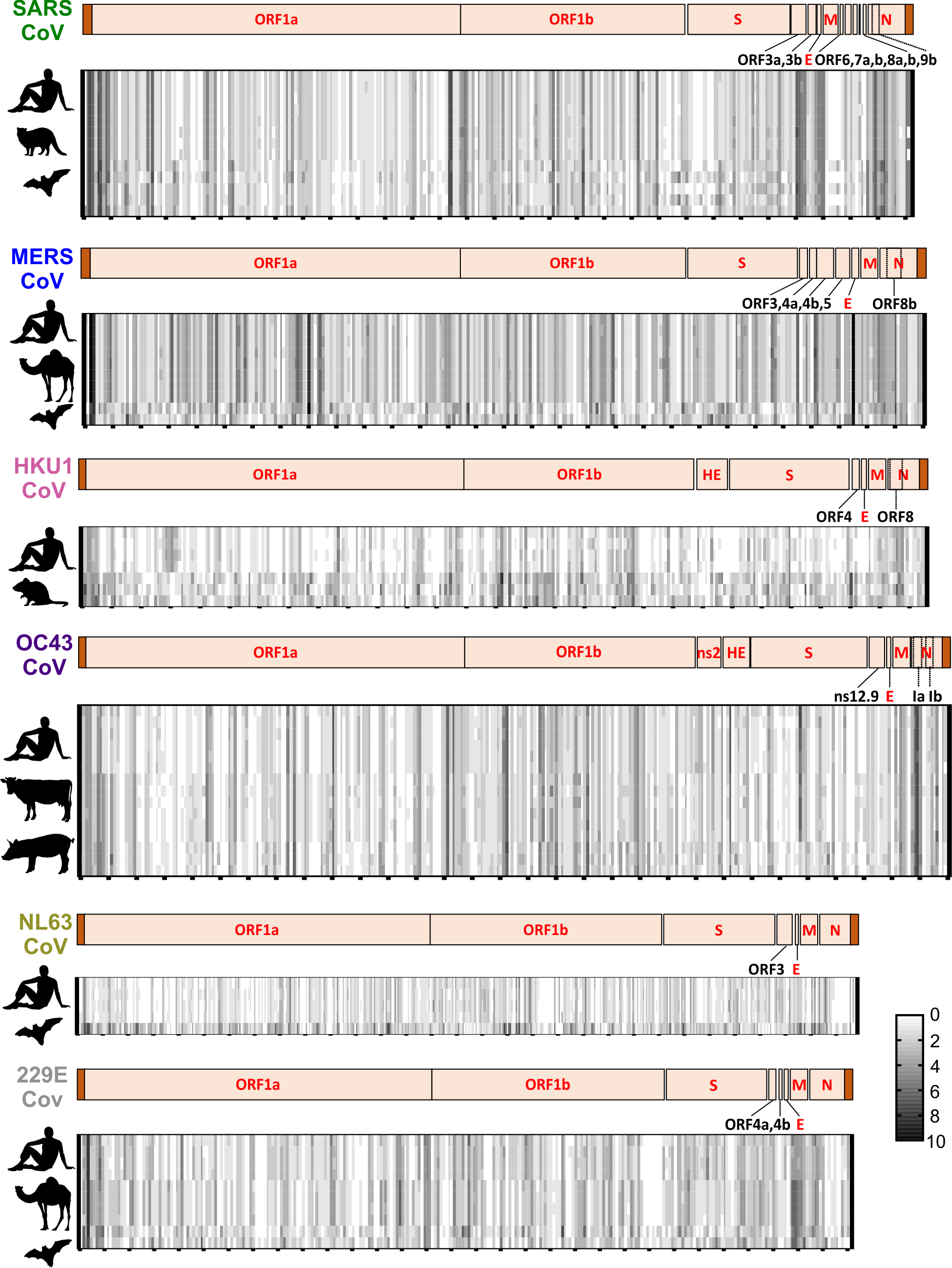

Supplement: FIG S2 [file mBio.01930-20-sf002.tif]

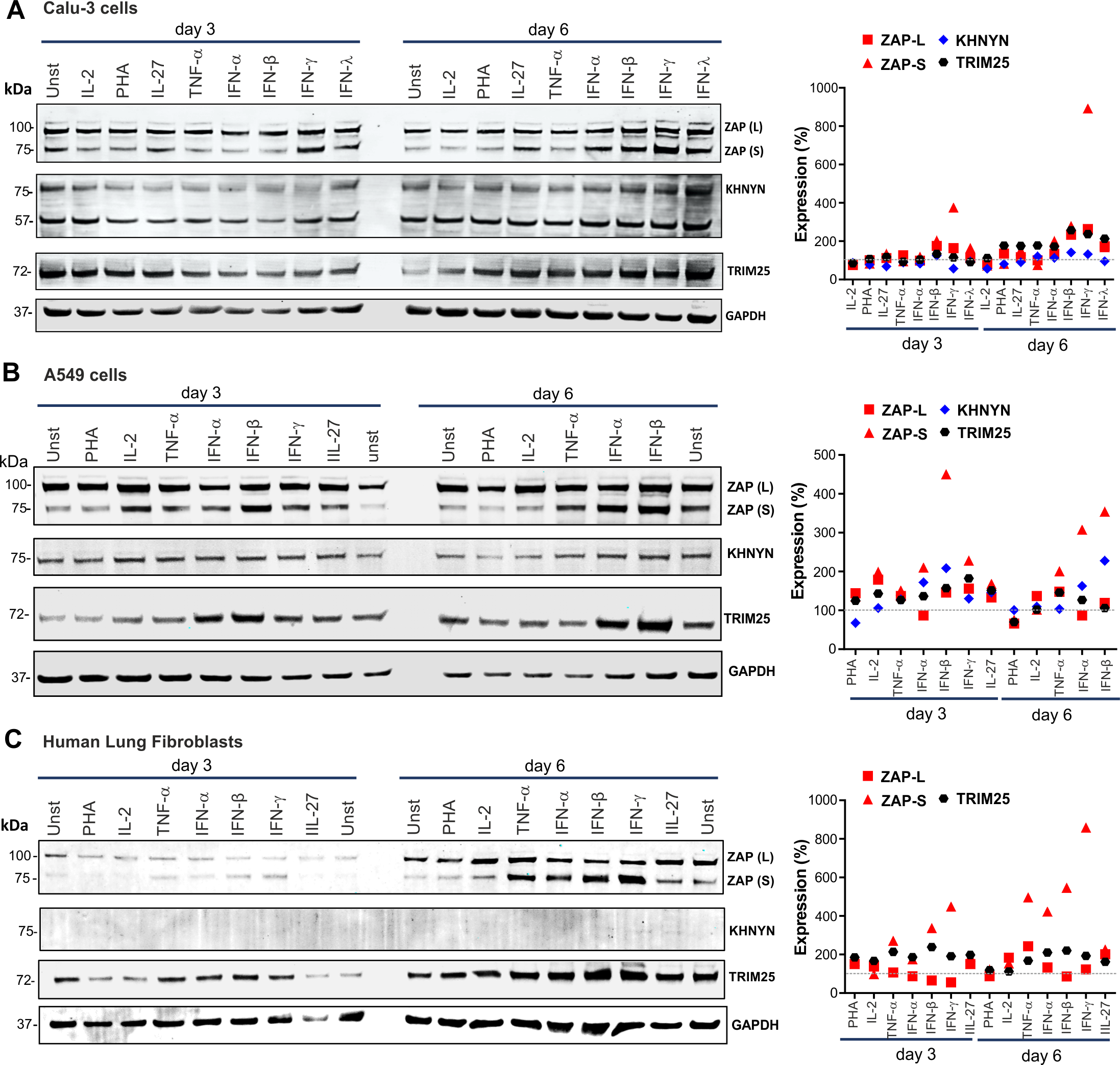

Supplement: FIG S3 [file mBio.01930-20-sf003.tif]

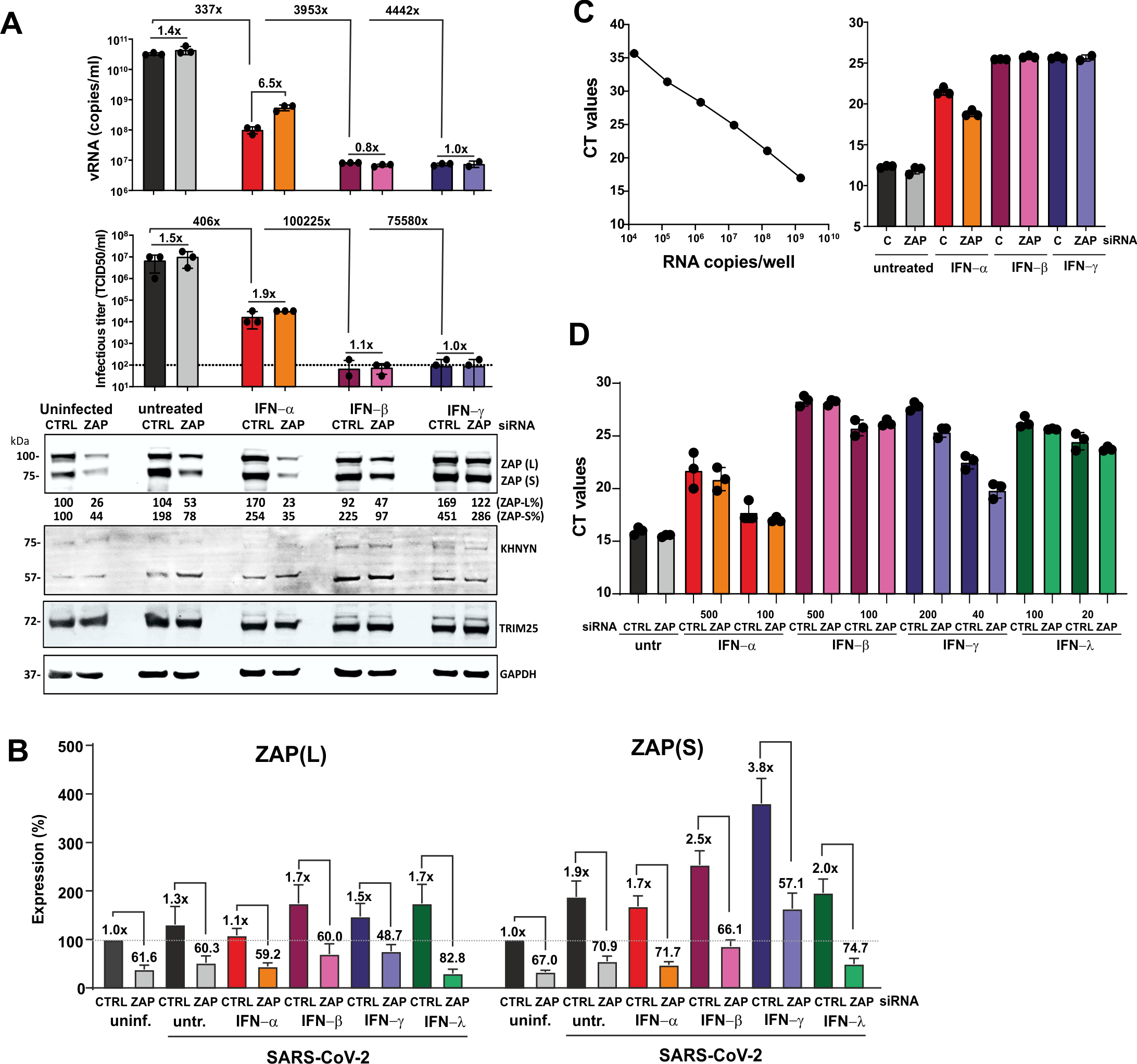

Supplement: FIG S4 [file mBio.01930-20-sf004.tif]
